# Beyond the scale: bioelectrical impedance for neonatal growth

**DOI:** 10.1038/s41390-026-04995-5

**Published:** 2026-04-21

**Authors:** Kimberley J. Botting-Lawford, Dino A. Giussani

**Affiliations:** 1 EGA Institute for Women’s Health, Faculty of Population Health Sciences, University College London, London, UK; 2 Physiology, Development & Neuroscience, University of Cambridge, Cambridge, UK; 3 Cambridge Cardiovascular Interdisciplinary Research Centre, University of Cambridge, Cambridge, UK; 4 Cambridge Reproduction Interdisciplinary Research Centre for, University of Cambridge, Cambridge, UK

Optimising growth in very preterm and very low birth weight infants remains a central challenge in neonatal care, requiring clinical decisions that balance survival, maturation, and long-term developmental outcomes. Growth in this vulnerable population is traditionally assessed using anthropometric measures, such as body weight and length. However, this offers limited insight into the underlying changes in body composition that accompany early postnatal adaptation and clinical interventions. Bioelectrical impedance analysis (BIA) is a promising alternative, providing a low-cost, minimally invasive approach that is well-suited to the neonatal intensive care unit (NICU) and enables repeated, longitudinal assessment of body composition.^1^ By capturing dynamic changes in fat mass (FM) and fat-free mass (FFM), BIA offers an opportunity to improve understanding of how treatments such as corticosteroids, alongside nutrition and other therapeutic exposures, shape neonatal growth trajectories in very preterm and very low birth weight infants, beyond what can be inferred from size-based measures alone.

**Fig. 1:**
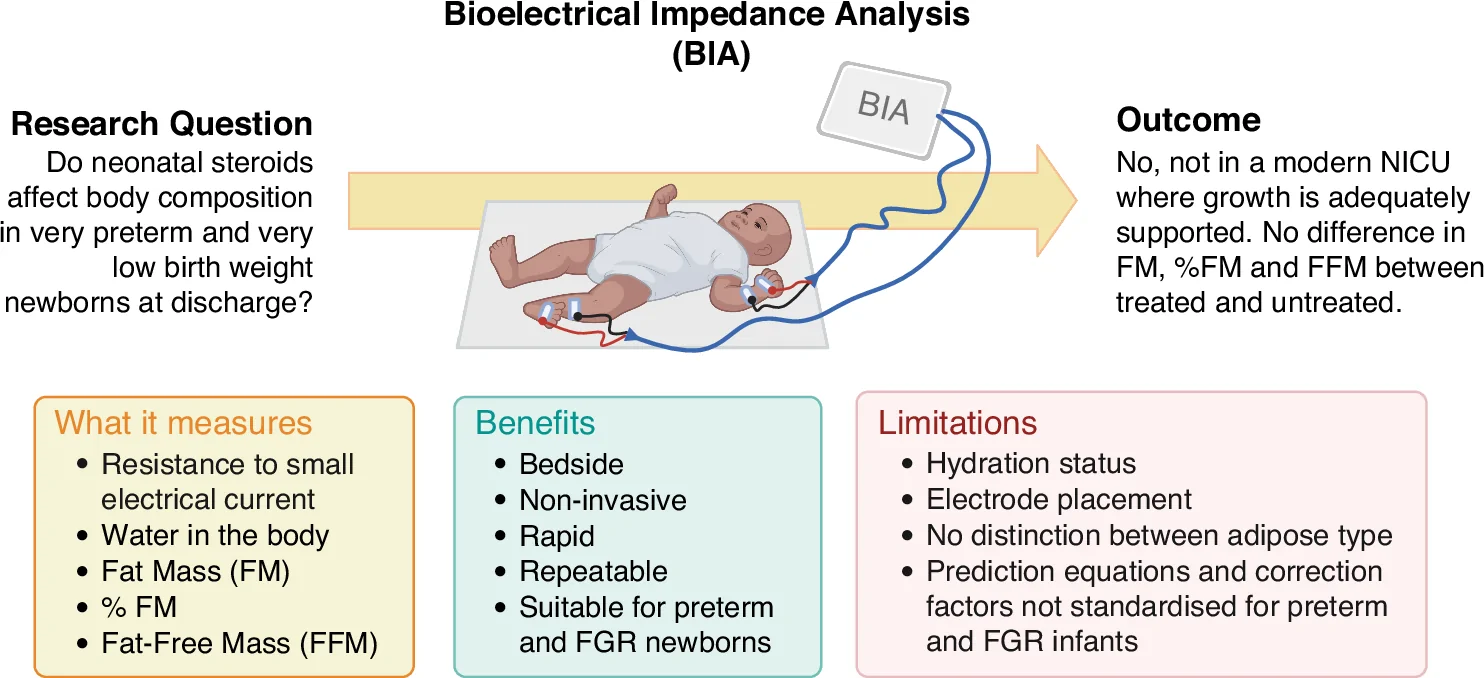
Bioelectrical impedance analysis (BIA) in very preterm and very low birth weight infants. Kraemer et al. used BIA to measure body composition in 62 very preterm or very low birth weight infants at discharge and found that neonatal corticosteroids do not affect fat mass (FM) or fat-free mass (FFM). Foetal growth restriction (FGR); Image created using BioRender.

The recently published prospective single-centre study by Kraemer and colleges,^2^ used BIA to assess body composition at hospital discharge among very preterm or low birth weight infants that were either exposed or not exposed to neonatal corticosteroids (Fig. 1). With 62 infants born <32 weeks of gestation or with a birth weight <1500 g, this study represents the first investigation of its kind to use BIA to address the effect of steroids on postnatal growth. The authors conclude that at hospital discharge neonatal corticosteroid exposure did not significantly alter FM, %FM, or FFM. Given the implications for both clinical care and the mechanistic understanding of corticosteroid effects, the findings merit critical examination.

Accurate and feasible assessment of body composition in preterm infants is challenging. Gold standard methods such as air displacement plethysmography (ADP) and dual-energy X-ray absorptiometry (DXA) provide robust measurements of FM, FFM and total body water. However, these are limited by cost, requirement for specialised equipment, and logistic constraints in the NICU. Conversely, traditional anthropometric and skinfold measurements are quick and inexpensive but lack precision in estimating compartmental body composition.^3^ BIA estimates body composition by measuring opposing properties to an applied alternating electrical current as it passes through body tissues. Because lean tissues contain a high proportion of water and electrolytes, they conduct electrical current more effectively than adipose tissue, which contains relatively little water. By quantifying resistance and reactance, BIA allows estimation of total body water, from which FFM mass can be calculated, and FM subsequently derived using validated predictive equations.^4^ However, BIA has recognised methodological considerations, as its accuracy can be influenced by hydration status, electrode placement, prediction equations, gestational age, and the dynamic body water distribution characteristic of early life physiology.^3^ In the present study, electrodes were placed on the hands and feet, thereby capturing the composition of the limbs and torso, while cranial tissue was not included.^2^ Evidence on the effects of neonatal corticosteroids on cerebral growth in preterm infants remains mixed, with reports of both increased and reduced cerebral growth, measured by MRI and ultrasound, respectively.^5,6^ Additionally, BIA cannot differentiate adipose tissue subtypes, limiting insight into the relative contributions of brown thermogenic, white metabolic, and immune-active adipose tissue, which requires advanced imaging modalities, including MRI or PET-CT. Growing evidence indicates that, when compared with the reference method of ADP, BIA can provide robust and clinically meaningful estimates of body composition in very low birth weight infants, particularly when appropriate, population-specific prediction equations and bias-correction factors are applied.^1^ While earlier work suggested that body weight alone outperformed BIA for predicting FFM,^7^ subsequent methodological advances and validation studies have strengthened confidence in BIA-derived measures. Consequently, BIA represents a valuable and practical tool for neonatal body composition assessment, with results optimally interpreted within the context of method specific validation and an understanding of its physiological and technical considerations.

Clinicians routinely administer postnatal corticosteroids to preterm infants at risk of bronchopulmonary dysplasia or prolonged ventilator dependence to reduce pulmonary morbidity,^8^ and to treat hypotensive shock and adrenal insufficiency. However, glucocorticoids such as dexamethasone and hydrocortisone have been linked with adverse neurodevelopmental and cardiovascular side-effects and possible slowing of somatic growth.^6,9–12^ Early postnatal growth and body composition are key determinants of lifelong lean mass, fat distribution, and metabolic health, and patterns established in infancy may shape future cardiometabolic risk.^13–15^ Therefore, understanding how neonatal corticosteroids affect body composition and these developmental processes is clinically important.

In the study by Kraemer and colleges,^2^ neonatal corticosteroid exposure was not associated with significant differences in total FM or FFM at discharge. This is consistent with contemporary clinical data indicating that, after adjustment for factors such as gestational age and severity of lung disease, postnatal corticosteroids do not significantly worsen weight gain, length, or head circumference during neonatal admission.^16,17^ In contrast, animal models show consistent growth impairment and complex, tissue‑specific effects on metabolism and body composition following glucocorticoid exposure.^18–21^ Against this background, BIA may represent a useful, low‑cost tool for longitudinal assessment of body composition across the life course in offspring born preterm.

In the current study, prematurity and foetal growth status were not examined as distinct contributors to postnatal body composition, which provides an important foundation for future investigation. It is plausible that the impact of neonatal corticosteroid exposure on lean and FM accrual may differ according to underlying foetal growth history and associated metabolic programming. Accordingly, future studies that incorporate FGR stratification will be well positioned to determine whether the effects of neonatal corticosteroids on body composition are modified by foetal growth patterns, thereby informing personalised therapeutic strategies.

In summary, Kraemer et al. ^2^ demonstrate that BIA is a feasible method for assessing body composition at discharge in very preterm infants and can be integrated into NICU research protocols. Their finding of no significant body composition differences between steroid-exposed and unexposed infants is preliminarily reassuring. However, widespread use of BIA as an outcome measure will require further validation of population-specific equations against reference methods (ADP, DXA) across diverse gestational ages and fluid states, as well as standardisation of measurement protocols (electrode placement, timing around fluid shifts, hydration correction). Future longitudinal studies should also be encouraged to examine whether early steroid exposure influences later adiposity, lean mass, or metabolic health, and should stratify by FGR and other pregnancy-related risk factors that may modify growth and body composition trajectories.
